# Phenotyping community-acquired pneumonia according to the presence of acute respiratory failure and severe sepsis

**DOI:** 10.1186/1465-9921-15-27

**Published:** 2014-03-04

**Authors:** Stefano Aliberti, Anna Maria Brambilla, James D Chalmers, Catia Cilloniz, Julio Ramirez, Angelo Bignamini, Elena Prina, Eva Polverino, Paolo Tarsia, Alberto Pesci, Antoni Torres, Francesco Blasi, Roberto Cosentini

**Affiliations:** 1 Clinica Pneumologica, Department of Health Science, University of Milan Bicocca, AO San Gerardo, Via Pergolesi 33, Monza, Italy; 2 Emergency Medicine Department, IRCCS Fondazione Ca’ Granda, Ospedale Maggiore Policlinico, Via F. Sforza 35, Milan, Italy; 3 Tayside Respiratory Research Group, Ninewells Hospital and Medical School, University of Dundee, Dundee, Scotland, UK; 4 Hospital Clínic, IDIBAPS, Ciberes, Barcelona, Spain; 5 Division of Infectious Diseases, Department of Medicine, University of Louisville, Louisville, KY, USA; 6 School of Specialization in Hospital Pharmacy, University of Milan, Via Colombo, 71, Milan, Italy; 7 Department of Pathophysiology and Transplantation, IRCCS Fondazione Ca’ Granda Ospedale Maggiore Policlinico, University of Milan, Via F. Sforza 35, Milan, Italy

**Keywords:** Pneumonia, Sepsis, Severe sepsis, Acute respiratory failure, ARDS, CAP, Community-acquired pneumonia, Mortality, Oxygenation

## Abstract

**Background:**

Acute respiratory failure (ARF) and severe sepsis (SS) are possible complications in patients with community-acquired pneumonia (CAP). The aim of the study was to evaluate prevalence, characteristics, risk factors and impact on mortality of hospitalized patients with CAP according to the presence of ARF and SS on admission.

**Methods:**

This was a multicenter, observational, prospective study of consecutive CAP patients admitted to three hospitals in Italy, Spain, and Scotland between 2008 and 2010. Three groups of patients were identified: those with neither ARF nor SS (Group A), those with only ARF (Group B) and those with both ARF and SS (Group C) on admission.

**Results:**

Among the 2,145 patients enrolled, 45% belonged to Group A, 36% to Group B and 20% to Group C. Patients in Group C were more severe than patients in Group B. Isolated ARF was correlated with age (p < 0.001), COPD (p < 0.001) and multilobar infiltrates (p < 0.001). The contemporary occurrence of ARF and SS was associated with age (p = 0.002), residency in nursing home (p = 0.007), COPD (p < 0.001), multilobar involvement (p < 0.001) and renal disease (p < 0.001). 4.2% of patients in Group A died, 9.3% in Group B and 26% in Group C, p < 0.001. After adjustment, the presence of only ARF had an OR for in-hospital mortality of 1.85 (p = 0.011) and the presence of both ARF and SS had an OR of 6.32 (p < 0.001).

**Conclusions:**

The identification of ARF and SS on hospital admission can help physicians in classifying CAP patients into three different clinical phenotypes.

## Background

Community-acquired pneumonia (CAP) is widely recognized as a major cause of morbidity and mortality [1]. Clinically, CAP exhibits an extreme variety in the severity of presentation, from almost asymptomatic disease at one side to a fulminant event to the other [2,3].

From a pathophysiological point of view, pneumonia can be complicated by the occurrence and interaction of two processes. On one hand, alveolar inflammation may result in serious ventilation-perfusion mismatch with the development of acute respiratory failure (ARF) that is shown to be associated, even in patients with mild pneumonia, with worse clinical outcomes [4]. On the other hand, decompartmentalization of the infection along with uncontrolled systemic inflammatory response might lead to the development of severe sepsis (SS). These two processes are linked together since interaction between hypoxia and inflammation has been proven at both molecular and cellular levels [5].

The natural history of pneumonia could be seen as a *continuum* from a local disease with neither respiratory nor multi-organ failure to a local disease leading to isolated respiratory failure to a systemic disease involving both respiratory failure and severe sepsis. If this were true, we would expect to see different clinical outcomes according to this three-stage model.

In order to test this hypothesis, we decided to evaluate prevalence, characteristics, risk factors, and impact on clinical outcomes of CAP patients with neither ARF nor SS, those with only ARF, and those with both ARF and SS on hospital admission.

## Methods

### Study design and setting

This was a multicenter, observational, prospective study of consecutive patients coming from the community who were admitted to the Policlinico Hospital (Milan, Italy), Hospital Clinìc (Barcelona, Spain) and NHS Hospitals (Edinburgh, UK) with diagnosis of pneumonia between April 2008 and April 2010. The Institutional Review Boards of all the hospitals approved the study. Patients **>**18 years of age who satisfied the criteria for pneumonia were included in the study. The following subjects were excluded: a) patients who were hospitalized in the previous 15 days; b) patients with a diagnosis of active tuberculosis or infection with fungi; c) immunosuppressed patients as those with HIV infection, neutropenia, on immunosuppressive therapy, chemotherapy, transplantation, cytotoxic therapy, and chronic systemic steroid therapy.

The following data were recorded: demographics; past medical history; severity of symptoms on admission; pneumonia severity index (PSI) and CURB-65 score; physical, laboratory, and radiological findings on admission; microbiological data; empiric antibiotic therapy; in-hospital mortality [6,7]. Blood gas analysis on admission was performed based on local standard operating procedures.

### Study definitions

Pneumonia was defined as the presence of a new pulmonary infiltrate on chest radiograph at the time of hospitalization associated with one or more of the following: (1) new or increased cough with/without sputum production; (2) fever (**> =** 37.8°C) or hypothermia (**<** 35.6°C); or (3) abnormal white blood cell count (either leukocytosis or leukopenia), or C-reactive protein values above the local upper limit.

Acute respiratory failure was defined as the presence of at least one among the following on admission: 1) partial pressure of oxygen in arterial blood (PaO_2_) < 60 mmHg; 2) ratio of PaO_2_ and fraction of inspired oxygen (PaO_2_/FiO_2_) < 300; 3) oxygen saturation < 90%; 4) respiratory acidosis, and 5) ventilatory support. Respiratory acidosis was considered when a pH value on admission of less than 7.35 was identified with a partial pressure of carbon dioxide in arterial blood (PaCO_2_) ≥ 45 mmHg.

Severe sepsis was defined as the presence of at least one of the following signs of organ hypoperfusion or organ dysfunction on admission: 1) sepsis-induced hypotension; 2) lactate greater than 2 mmol/L; 3) urine output <0.5 mL/kg hr for >2 hours; 4) creatinine >2.0 mg/dL; 5) bilirubin >2 mg/dL; platelet count <100,000 cell/L^−1^; 6) coagulopathy (international normalized ratio >1.5), as previously reported [8].

### Microbiology and empiric antibiotic therapy

Microbiological examinations were performed on sputum, urine, and blood during the first 24 hours after admission and according to standards of practice [9]. Empiric antibiotic therapy was administered as soon as the diagnosis of pneumonia was reached in the emergency department. The empiric antibiotic treatment was evaluated for compliance with the European Respiratory Society guidelines [10].

### Study groups and outcomes

Among the entire study population three groups of patients were identified based on the presence of ARF and SS on hospital admission: those with neither ARF nor SS (Group A), those with only ARF (Group B), and those with both ARF and SS (Group C) on hospital admission. Each single case of patients with a PaO_2_/FiO_2_ ratio between 300 and 315 and those with a creatinine more than 2 mg/dL on admission in the presence of chronic renal failure who could not be categorized into either group (n = 105) were reviewed by a clinical committee composed of two pulmonary (SA and FB) and one infectious disease physician (JR). After a comprehensive evaluation of all the available information, the committee was able to assign all the patients to one of the three study groups. In-hospital mortality and length of stay in the hospital (LOS) were the study outcomes. LOS was calculated as the number of days from the date of admission to the date of discharge.

### Statistical analysis

All data were statistically analyzed using SPSS (version 18.0) for Mac. Descriptive statistics were reported at baseline, with continuous data expressed as a median (25–75 interquartile range -IQR) and categorical data expressed as counts. Patient characteristics were compared between groups. Differences of continuous data between two groups were evaluated by Mann–Whitney *U* test (two groups) or Kruskal-Wallis test (three groups). Differences of categorical variables between two or more groups were analyzed using the *X*^2^ test or Fisher exact test where appropriate. The center effect on mortality was tested using a meta-analytical approach run in R [11,12]. Potential predictors of an adverse event that were considered of clinical relevance and immediately accessible on admission were investigated with the multivariable binomial logistic regression analysis and included: sex, age, comorbidities (diabetes, congestive heart failure, cardiovascular diseases, COPD, liver disease), nursing home residency, multilobar infiltrate and pleural effusion. The cumulative probability of survival over 14 days was tested with a Kaplan-Mayer analysis. In order to detect the pneumonia-related mortality, patients who died after 14 days from admission were considered to be alive for the purpose of the Kaplan-Mayer analysis. The reliability of the obtained results was also tested adjusting the survival analysis by center and confounders that were considered of clinical relevance and immediately accessible on admission using the Cox analysis. A p value <0.05 was considered statistically significant and was adjusted for multiplicity according to the Bonferroni criterion.

## Results

### Prevalence, characteristics, and risk factors for ARF and SS

A total of 2,145 consecutive patients with pneumonia were enrolled during the study period: 47% were males and median (IQR) age was 73 (56–82) years. Data to define either ARF or SS on admission were not available in 367 patients. A sensitivity analysis was performed checking whether the main outcome was different between the three circumstances: excluding patients with information not recorded; attributing missing information to non-ARF and/or non-SS; attributing missing information to ARF and/or SS. The three analyses yielded superimposed results (confidence intervals completely overlapping). Consequently, it was assumed that non-recorded data were in the normal range, and these patients were classified as non-ARF and/or non-SS.

Among the entire study population, 954 (45%) patients had neither ARF nor SS on admission (Group A). The presence of ARF alone was identified in 771 (36%) patients (Group B) and of both ARF and SS on admission in 420 (20%) patients (Group C). No patients with SS and without ARF were identified. Among patients with SS, the frequencies of organ failure were as follows: sepsis-induced hypotension in 204 (49%), creatinine >2.0 mg/dL in 140 (33%), urine output <0.5 mL/kg hr for >2 hours in 84 (20%), lactate greater than 2 mmol/L in 69 (16%), platelet count <100,000 cell/L^−1^ in 45 (11%), bilirubin >2 mg/dL in 36 (8.6%) and coagulopathy in 26 (6.2%) patients. Demographics, severity of disease, clinical, laboratory, and radiological findings on admission, microbiology and empiric antibiotic therapy of the three study groups are summarized in Table 1. In comparison to patients with ARF alone, those with both ARF and SS on admission had more comorbidities, a more diffuse radiological involvement (p = 0.003), were more hypoxemic (p < 0.001) and with a higher proportion of patients on respiratory acidosis (p = 0.014).

**Table 1:** Demographics, severity of disease, clinical, laboratory, radiological findings on admission, microbiology and empiric antibiotic therapy of the study population, according to the three study groups

**Characteristic**	**Group A**	**Group B**	**Group C**	**p (Among 3 groups)**	**p (Group B vs. Group C)**
n. (%)	954 (100)	771 (100)	420 (100)		
**Demographics**					
Male, n. (%)	451 (47)	368 (48)	186 (44)	0.492	0.255
Age, median (IQR) years	67 (47–81)	75 (61–83)	75 (61–84)	<0.001	0.868
**Comorbidities, n. (%)**					
Congestive heart failure	148 (16)	170 (22)	103 (25)	<0.001	0.332
Chronic obstructive pulmonary disease	139 (15)	237 (31)	115 (27)	<0.001	0.225
Diabetes mellitus	111 (12)	118 (15)	61 (15)	0.069	0.731
Cerebrovascular disease	83 (9)	93 (12)	70 (17)	<0.001	0.027
Chronic renal failure	63 (7)	63 (8)	68 (16)	<0.001	<0.001
Liver disease	52 (6)	27 (4)	32 (8)	0.008	0.002
Residency in a nursing home	46 (5)	63 (8)	54 (13)	<0.001	0.009
**Severity on admission, n. (%)**					
PSI Risk Class IV and V	332 (35)	464 (60)	343 (82)	<0.001	<0.001
CURB-65 score 3, 4 and 5	127 (13)	198 (26)	231 (55)	<0.001	<0.001
Admission to ICU	6 (0.6)	54 (7)	90 (21)	<0.001	<0.001
**Physical findings on admission, median (IQR)**					
Systolic blood pressure, mmHg	127 (112–145)	130 (115–150)	108 (85–133)	<0.001	<0.001
Diastolic blood pressure, mmHg	70 (64–80)	71 (62–80)	60 (50–72)	<0.001	<0.001
Heart rate, beats/minute	95 (83–107)	100 (85–114)	110 (100–120)	<0.001	<0.001
Respiratory rate, breaths/minute	20 (18–26)	24 (20–30)	30 (24–34)	<0.001	<0.001
SpO_2_,%	96 (95–97)	92 (89–95)	90 (85–93)	<0.001	<0.001
**Laboratory values, median (IQR)**					
Arterial pH	7.45 (7.42-7.48)	7.44 (7.40-7.48)	7.39 (7.31-7.46)	<0.001	<0.001
PaO_2_/FiO_2_ ratio	339 (318–378)	256 (216–279)	229 (182–276)	<0.001	<0.001
PaCO_2_, mmHg	33 (29–38)	36 (31–42)	36 (30–48)	<0.001	0.164
Respiratory acidosis, n. (%)	0 (0)	59 (8)	47 (13)	<0.001	0.014
White blood cells, cell/L^−1^	11900 (8238–16300)	12790 (9100–17000)	144000 (9993–19000)	<0.001	0.001
Platelet, cell/L^−1^	232000 (182000–300500)	237000 (186500–317000)	233000 (166750–307250)	0.054	0.028
Hemoglobin, g/dL	13 (12–14)	13 (12–14)	13 (11–14)	0.010	0.021
Hematocrit,%	39 (36–42)	40 (37–44)	39 (34–43)	<0.001	0.001
Urea, mg/dL	37 (27–54)	45 (31–64)	63 (42–103)	<0.001	<0.001
Creatinine, mg/dL	0.9 (0.8-1.2)	1.0 (0.8-1.2)	1.2 (0.9-1.9)	<0.001	<0.001
Sodium, mEq/L	137 (134–139)	137 (134–139)	136 (133–140)	0.650	0.409
Glucose, mg/dL	105 (73–128)	113 (74–148)	101 (65–149)	0.002	0.035
**Radiology findings on CXR, n. (%)**					
Multilobar involvement	161 (18)	195 (28)	141 (36)	<0.001	0.003
Pleural effusion	204 (22)	150 (20)	100 (24)	0.209	0.078
**Microbiological findings, n. (%)**					
Patients with isolated bacteria	184 (19)	198 (26)	141 (34)	<0.001	0.004
Polymicrobial infection	3 (2)	8 (4)	4 (3)	0.369	0.547
Patients with > = one MDR pathogen	17 (4)	20 (5)	22 (8)	0.015	0.060
*S. pneumoniae*	90 (49)	114 (58)	64 (45)	0.064	0.027
*S. aureus*	15 (8)	19 (10)	23 (16)	0.049	0.064
*Legionella pneumophila*	16 (9)	16 (8)	5 (4)	0.156	0.088
Respiratory viruses	11 (6)	12 (6)	13 (9)	0.439	0.273
Atypicals	31 (17)	22 (11)	9 (6)	0.014	0.137
*H. influenzae*	17 (9)	14 (7)	7 (5)	0.328	0.419
**Empiric antibiotic therapy, n. (%)**					
Levofloxacin	287 (30)	234 (30)	106 (25)	0.133	
Ceftriaxone	348 (37)	324 (42)	145 (35)	0.015	
Amoxicillin (clavulanate)	295 (31)	218 (28)	148 (35)	0.045	
Azithromycin	230 (24)	189 (25)	72 (17)	0.007	
Clarithromycin	198 (21)	211 (27)	152 (36)	<0.001	
Piperacillin/tazobactam	34 (3.6)	44 (5.7)	43 (10)	<0.001	
Antibiotics compliant with local guidelines	731 (81)	595 (79)	328 (79)	0.606	0.933
**Outcomes**					
Length of hospital stay, median (IQR) days	6 (3–11)	9 (6–14)	12 (7–21)	<0.001	0.004
In-hospital mortality, n. (%)	43 (4.2)	72 (9.3)	108 (26)	<0.001	<0.001

n: number; IQR: 25–75 interquartile range; COPD: chronic obstructive pulmonary disease; CAP: community-acquired pneumonia; PSI: pneumonia severity index; ICU: intensive care unit; CXR: chest radiograph; ESBL: extended-spectrum beta-lactamase; MDR: multidrug resistant (including methicillin-resistant *S. aureus*, *Pseudomonas aeruginosa* resistant to antipseudomonal penicillins, cephalosporins, carbapenems, and quinolones, *Stenotrophomonas maltophilia*, vancomycin-resistant *Enterococcus*, *Acinetobacter baumanii*, extended spectrum b-lactamase producing *Enterobacteriaceae,* and other non-fermenting Gram-negative bacilli); SpO_2_: oxygen saturation; PaO_2_: partial pressure of oxygen in arterial blood; PaCO_2_: partial pressure of carbon dioxide in arterial blood. ^+^Hypotension defined as Systolic blood pressure <90 mm Hg or diastolic blood pressure < 60 mm Hg. Differences in continuous variables were monitored with Kruskal-Wallis test (three groups) and Mann–Whitney *U* Test differences (two groups); differences in proportions were monitored with ANOVA (three groups) and the chi square test (two groups).

Group A: Community-acquired pneumonia (CAP) patients with neither acute respiratory failure (ARF) nor severe sepsis (SS) on admission. Group B: CAP patients with only ARF on admission. Group C: CAP patients with both ARF and SS on admission.

Centers, demographics, comorbidities, and radiological findings were tested in multivariate logistic regression as potential predictors of outcome, and age (p < 0.001), the presence of COPD (p < 0.001), and multilobar infiltrates on CXR (p < 0.001) resulted significantly associated with isolated ARF on admission. Centers, demographics, comorbidities, and radiological findings were tested in multivariate logistic regression as potential predictors of outcome, and age (p = 0.002), residency in nursing home (p = 0.007), the presence of COPD (p < 0.001), multilobar involvement (p < 0.001), and renal disease (p < 0.001) resulted significantly associated with the contemporary occurrence of ARF and severe sepsis on admission, see Table 2.

**Table 2:** Risk factors associated to the presence of isolated acute respiratory failure and both acute respiratory failure and severe sepsis on admission in the study population

	**Only acute respiratory failure on admission**		**Both acute respiratory failure and severe sepsis on admission**	
**Age**	1.02 (1.01-1.03)	<0.001	1.01 (1.01-1.02)	0.002
**Chronic obstructive pulmonary disease**	2.08 (1.60-2.70)	<0.001	1.85 (1.34-2.56)	<0.001
**Multilobar infiltrate**	1.96 (1.51-2.55)	<0.001	3.08 (2.26-4.19)	<0.001
**Pre-existing renal disease**			2.18 (1.42-3.35)	<0.001
**Residency in nursing home**			1.94 (1.20-3.14)	0.007
**Female sex**			0.74 (0.57-0.96)	0.025

Data represent odds ratio with 95% confidence intervals at multivariable binomial logistic regression analysis.

### The impact of ARF and SS on mortality

**Figure 1:**
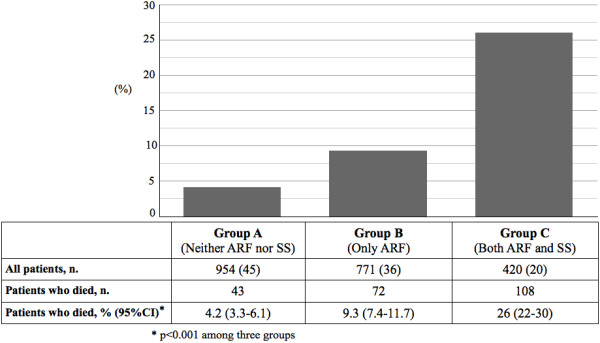
**In-hospital mortality of the study population according to the three study groups.** Group A: Community-acquired pneumonia (CAP) patients with neither acute respiratory failure (ARF) nor severe sepsis (SS) on admission. Group B: CAP patients with only ARF on admission. Group C: CAP patients with both ARF and SS on admission. n: number.

**Figure 2:**
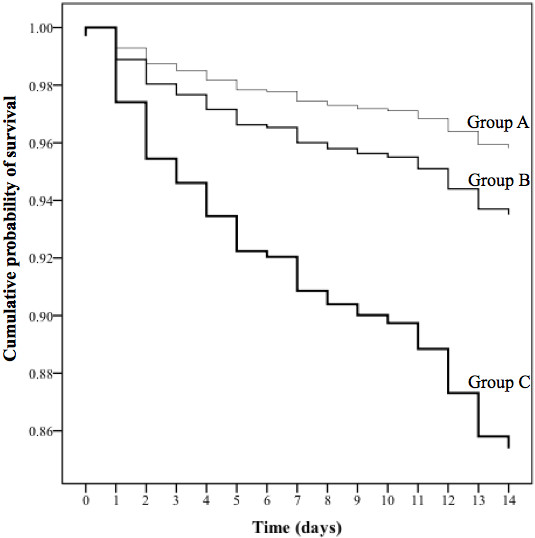
**The cumulative probability of survival in the three study groups.** Group A: community-acquired pneumonia (CAP) patients with neither acute respiratory failure (ARF) nor severe sepsis (SS) on admission. Group B: CAP patients with only ARF on admission. Group C: CAP patients with both ARF and SS on admission. Adjusted for centers, sex, age, comorbidities (including congestive heart failure, cerebrovascular accident, diabetes, chronic obstructive pulmonary disease, liver and renal diseases), residence in nursing home, multilobar infiltrate, pleural effusion, empiric antibiotic therapy concordant with European respiratory Society guidelines.

A total of 223 patients (10%; 95% CI: 9.2%-11.8%) among the study population died during hospitalization. The meta-analysis of the absolute risk difference in mortality between the study groups failed to exhibit a significant heterogeneity by centers, see Additional file 1: Table S1 and Additional file 2: Figure S1. The in-hospital mortality of the study population is summarized in Figure 1 according to the three study groups. A total of 43 patients died in Group A (4.2%; 95% CI: 3.3%-6.1%), 72 patients in Group B (9.3%; 95% CI: 7.4%-11.7%), and 108 patients in Group C (26%; 95% CI: 22%-30%), p < 0.001. The survival analysis showed a significant difference in terms of time to death among the three study groups: 13.6 days (Group A) vs. 13.2 days (Group B), p = 0.005; 13.6 days (Group A) vs. 11.9 days (Group C), p < 0.001 (Log Rank test). The Cox survival analysis confirmed these results after adjusting for several confounders, see Figure 2.

**Figure 3:**
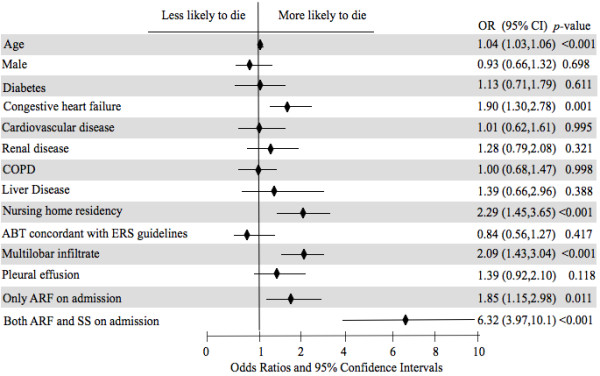
**Multivariable logistic model with respect to in-hospital mortality.** ARF: acute respiratory failure; SS: severe sepsis; COPD: chronic obstructive pulmonary disease; ABT: antibiotic empiric therapy; ERS: European Respiratory Society; OR: odds ratio; CI: confident intervals. All the variables are present vs. absent, but age (1-year change), male (vs. female) and multilobar (vs. monolobar).

Factors associated with in-hospital mortality in the study population at the univariate analysis are shown in Additional file 1: Table S1. At the multivariable logistic regression model (1,891 patients; Nagelkerke R^2^: 0.253; p from the Hosmer-Lemeshow test = 0.105), after adjustment for centers and several confounders, the presence of only ARF on admission had an OR for in-hospital mortality of 1.85 (95% CI: 1.15-2.98, p = 0.011) and the presence of both ARF and SS on admission had an OR of 6.32 (95% CI: 3.97-10, p < 0.001), see Figure 3. The presence of multilobar infiltrate on CXR showed an adjusted OR for mortality of 2.09 (95% CI: 1.43-3.04, p < 0.001).

**Figure 4:**
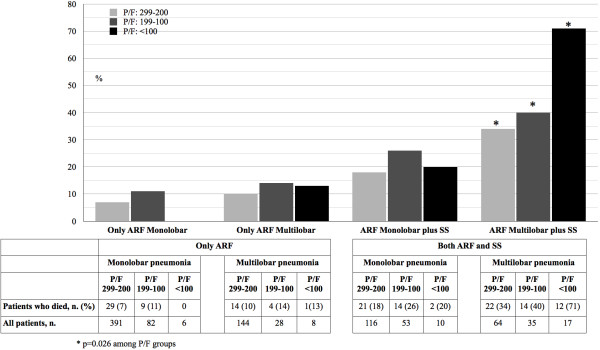
**In-hospital mortality in community-acquired pneumonia patients with only acute respiratory failure (ARF) on admission and with both ARF and severe sepsis (SS) on admission stratified by mono- or multilobar involvement on chest X-ray and PaO**_**2**_**/FiO**_**2 **_**ratio (P/F) levels on admission.**

Figure 4 summarizes the mortality in patients with isolated ARF on admission, according to PaO_2_/FiO_2_ ratio and the presence of multilobar infiltrate on CXR. No significant difference in mortality was detected in these patients (Group B) according to the presence of monolobar vs. multilobar involvement on CXR (38/479 patients, 7.9% vs. 19/180 patients, 11%, p = 0.350). No significant difference in mortality was observed in Group B among patients with different levels of PaO_2_/FiO_2_ ratio, regardless of multilobar involvement. Mortality in patients with both ARF and SS on admission is depicted in Figure 4, according to PaO_2_/FiO_2_ ratio and the presence of multilobar infiltrate on CXR. Mortality rate in these patients (Group C) was significantly different according to the presence of monolobar vs. multilobar involvement on CXR (37/179 patients, 21% vs. 48/116 patients, 41%, p < 0.001). No significant difference was observed among patients with different levels of PaO_2_/FiO_2_ ratio in the presence of monolobar involvement on CXR, whereas a significant difference was seen between different levels of PaO_2_/FiO_2_ ratio in the presence of multilobar involvement on CXR (p = 0.026).

## Discussion

In the present study we showed different characteristics on admission as well as significant differences in mortality among hospitalized patients with CAP according to the presence of ARF and SS on admission. Three distinct groups of CAP patients have been identified: those with neither ARF nor SS (4% mortality), those with only ARF (9% mortality), and those with both ARF and SS on admission (26% mortality). After adjustment, ARF alone on admission has an OR for mortality of 1.84 and the association of ARF and SS increases the OR for mortality up to 6.55. Furthermore, the evaluation of the presence of multilobar infiltrate on CXR on admission can help in better stratifying CAP patients according to their risk of death.

A lack of data exists about the prevalence of ARF in a general population of CAP patients admitted to the hospital. Furthermore, the characteristics of the patients enrolled in previous studies are very different from each other, as well as definitions of ARF [3,13-15]. Although all these factors make it difficult to compare the results, the prevalence of ARF reported in previous literature ranges between 16 to 73%, mostly in line with the 56% of prevalence detected in our study. SS represents a common complication in CAP patients, involving up to 34% of patients, and it seems to be acquired during the first days of hospitalization [16,17]. The prevalence of SS rises up to 67% in patients with ARF who are admitted to the ICU for different diseases [18]. Our data are in line with previous experiences showing the presence of SS on admission in 20% of hospitalized patients with CAP.

We identified three different groups of patients with CAP based on the absence of both ARF and SS, the presence of ARF alone, and the presence of both ARF and SS. In view of the differences between patients belonging to these three groups in terms of both baseline characteristics and mortality, we could speculate that three distinct clinical phenotypes of CAP patients exist. Furthermore, we have shown that the evaluation of the presence of multilobar involvement on admission on CXR can help in stratifying patients according to in-hospital mortality. A recent meta-analysis by Mannu and coworkers showed that multilobar involvement is an independent risk factor for mortality [19]. We observed the highest in-hospital mortality among CAP patients with ARF, severe sepsis and multilobar infiltrates. Furthermore, we were able to unmask an effect of gas exchange (PaO_2_/FiO_2_ ratio) on in-hospital mortality only in this group of patients. Multilobar involvement is a well-known reflection of the severity of the CAP and could indicate either a direct involvement of the microbial challenge or an indirect involvement in the context of acute respiratory distress syndrome (ARDS). Based on our results, it could be suggested that in patients with both severe sepsis and ARF due to a multilobar CAP, the lung should be considered as the starting point of a systemic inflammatory response such as for ARDS and not just as an organ affected by a single infectious process.

The findings of the present study could lead to some speculations from both a clinical and a research point of view. Clinically, it is widely accepted that an early and correct identification of patients at risk of death is a crucial step in the management of CAP patients and several indices to predict mortality have been developed [20]. Although these scores have been validated in the scientific literature over the last 20 years, it seems they have serious difficulties to be implemented in daily clinical practice and that they are inconsistent with clinical judgment in a significant percentage of patients in low risk classes [21-23]. Furthermore, it has been recently shown that the CURB-65, the most simple tool suggested by international guidelines to decide hospitalization for CAP patients, suffers of a lack a formal assessment of hypoxemia, a major drawback in light of the importance of assessing oxygenation immediately on arrival at the ER [24,25]. Furthermore, no score in the decision to hospitalize patients in the ICU has been widely accepted in clinical practice [26,27]. Some authors have suggested a more pathophysiological and simple approach in the assessment of severity of CAP based on evaluation of the presence of ARF and SS [28]. The three-group classification of CAP patients we proposed could be useful in this sense and may help to build up a new algorithm for the site-of-care decision [29].

A meta-analysis of the data from our three study sites showed no heterogeneity in the risks associated with ARF and ARF/SS, validating that these are consistent, robust phenotypes. The presence of these three groups that clearly differ in characteristics and outcomes makes it necessary to search for different underlying biological and molecular processes. Previous data have shown a different genotype association for septic shock and hypoxemic ARF in CAP patients [30]. The identification of these three clinically different phenotypes would be an important guide in the interpretation of the large amount of information that will possibly come from the “-omics” world in next few years. The explanation at a basic level of these clinical findings could finally allow the development of new and interesting therapeutic measurements, especially in CAP patients with SS.

Our study has some limitations. We were not able to collect data concerning the response to fluid challenge in case of initial hypotension. Thus, patients with septic shock are included in the severe sepsis definition, although a higher mortality should be expected in these patients. Time to first antibiotic dose and treatment of ARF and SS during hospitalization were not evaluated in our study. However, recent data suggest that time to first antibiotic dose should be interpreted as marker of optimal care in CAP patients rather than a predictor of outcomes [31]. Furthermore, all three centers have standard operating procedure for CAP patients and ARF and SS are managed according to international guidelines.

Our study was strengthened by the evaluation of three large cohorts of consecutive, prospectively enrolled patients in three different regions in Europe in very large and robust data collections. The second main strength is that we described a population, easy to identify with clinical and laboratory variable collected at the emergency room, having a high probability to die, which is a target population to implement intensive treatment. This concept fits perfectly with the idea of CAP as a medical emergency [32].

## Conclusions

The foremost conclusion of our study is that the identification of ARF and SS on hospital admission can help physicians in classifying CAP patients into three different clinical phenotypes: those with neither ARF nor SS, those with only ARF and those with both ARF and SS. Since these three groups of patients show different characteristics and outcomes, this simple and intuitive classification could be used in the site-of-care decision for CAP patients with the goal to start as early as possible the appropriate treatment in the right setting.

### Consent

Written informed consent was obtained from the patient's guardian/parent/next of kin for the publication of this report and any accompanying images.

## Abbreviations

ARF: Acute respiratory failure; CAP: Community-acquired pneumonia; CI: Confidence interval; COPD: Chronic obstructive pulmonary disease; CXR: Chest X-ray; ERS: European respiratory society; ESBL: Extended-spectrum beta-lactamase; FiO2: Fraction of inspired oxygen; HIV: Human immunodeficiency virus; ICU: Intensive care unit; IQR: Interquartile range; LOS: Length of stay in the hospital; MDR: Multidrug resistant; NHS: National Health Service; OR: Odds ratio; PaCO2: Partial pressure of carbon dioxide in arterial blood; PaO2: Partial pressure of oxygen in arterial blood; PSI: Pneumonia severity index; SpO2: Oxygen saturation; SS: Severe sepsis.

## Competing interests

The authors declare that they have no competing interests.

## Authors’ contributions

Study concept and design: SA, RC, AMB. Acquisition of data: SA, AMB, JDC, CC, EvaP. Analysis and interpretation of data: SA, AMB, JDC, CC, JR, AB, EvaP, EP, PT, AP, AT, FB. RC. Drafting of the manuscript: SA, JR, AT, RC, AMB, FB. Critical revision of the manuscript for important intellectual content: All authors. Statistical analysis: SA, AB. Study supervision: AT, FB, RC. All authors read and approved the final manuscript.

## Supplementary Material

Additional file 1: Table S1 Demographics, severity of disease, clinical, laboratory, radiological findings on admission, microbiology and empiric antibiotic therapy of the study population, according to survival during hospitalization. Click here for file

Additional file 2: Figure S1 The meta-analysis of the absolute risk difference in mortality between the study groups. ARF: acute respiratory failure, SS: severe sepsis. Click here for file
