# Supplementary material for: Phenotyping community-acquired pneumonia according to the presence of acute respiratory failure and severe sepsis
Source: Respir Res. 2014 Mar 4;15(1):27. doi: 10.1186/1465-9921-15-27 (PMC4015148; doi:10.1186/1465-9921-15-27)
Supplement: Additional file 1: Table S1 — Demographics, severity of disease, clinical, laboratory, radiological findings on admission, microbiology and empiric antibiotic therapy of the study population, according to survival during hospitalization. [file 1465-9921-15-27-S1.doc]

**Additional file 1: Table S1. Demographics, severity of disease, clinical, laboratory, radiological findings on admission, microbiology and empiric antibiotic therapy of the study population, according to survival during hospitalization**

| **Characteristic** | **Survivors** | **Died** | p |
| --- | --- | --- | --- |
| n. (%) | 1,922 (100) | 223 (100) |  |
| **Demographics** |  |  |  |
| Male, n. (%) | 902 (47) | 103 (46) | 0.834 |
| Age, median (IQR) years | 70 (54-81) | 81 (72-88) | <0.001 |
| **Comorbidities, n. (%)** |  |  |  |
| Congestive heart failure | 333 (17) | 88 (40) | <0.001 |
| Chronic Obstructive Pulmonary Disease | 428 (22) | 63 (28) | 0.045 |
| Diabetes mellitus | 250 (13) | 40 (18) | 0.042 |
| Cerebrovascular disease | 194 (10) | 52 (23) | <0.001 |
| Chronic renal failure | 149 (7.8) | 45 (20) | <0.001 |
| Liver disease | 97 (5.1) | 14 (6.3) | 0.434 |
| Residency in a nursing home | 112 (5.8) | 51 (23) | <0.001 |
| **Severity on admission, n. (%)** |  |  |  |
| PSI Risk Class IV and V | 937 (49) | 202 (91) | <0.001 |
| CURB-65 score 3, 4 and 5 | 426 (22) | 130 (58) | <0.001 |
| Admission to Intensive Care Unit | 125 (6.6) | 30 (14) | <0.001 |
| **Physical findings on admission, median (IQR)** |  |  |  |
| Systolic blood pressure, mmHg | 126 (110-145) | 117 (98-141) | <0.001 |
| Diastolic blood pressure, mmHg | 70 (60-80) | 68 (60-80) | <0.001 |
| Heart rate, beats/minute | 100 (86-110) | 107 (92-120) | <0.001 |
| Respiratory rate, breaths/minute | 24 (20-30) | 28 (24-35) | <0.001 |
| SpO_2_, mean (SD) % | 94 (91-96) | 89 (85-95) | <0.001 |
| **Laboratory values, median (IQR)** |  |  |  |
| Arterial pH | 7.44 (7.40-7.48) | 7.38 (7.30-7.46) | <0.001 |
| PaO_2_, mmHg | 63 (56-73) | 56 (49-68) | <0.001 |
| PaO_2_/FiO_2_ ratio | 279 (236-320) | 232 (183-276) | <0.001 |
| PaCO_2_, mmHg | 35 (30-40) | 37 (30-48) | <0.001 |
| Respiratory acidosis | 102 (7) | 47 (24) | <0.001 |
| White blood cells, cell/L^-1^ | 12420 (9000-17000) | 13550 (9100-18000) | 0.174 |
| Platelet, cell/L^-1^ | 234000 (182000-307000) | 233000 (170000-303500) | 0.462 |
| Hemoglobin, g/dL | 13 (12-14) | 12.5 (11-14) | <0.001 |
| Hematocrit, % | 40 (36-43) | 39 (34-44) | 0.187 |
| Urea, mg/dL | 42 (29-60) | 78 (49-112) | <0.001 |
| Creatinine, mg/dL | 1 (0.8-1.2) | 1.2 (0.9-1.8) | <0.001 |
| Sodium, mEq/L | 137 (134-139) | 138 (133-141) | 0.007 |
| Glucose, g/dL | 106 (71-137) | 118 (76-161) | 0.018 |
| **Radiology findings on CXR, n. (%)** |  |  |  |
| Multilobar involvement | 411 (23) | 86 (45) | <0.001 |
| Pleural effusion | 388 (20) | 66 (30) | 0.001 |
| **Microbiological and empiric antibiotics, n. (%)** |  |  |  |
| Polymicrobial infection | 12 (2.6) | 3 (5.4) | 0.236 |
| Patients with >= one MDR pathogen | 47 (4.4) | 12 (11) | 0.003 |
| Compliant with ERS guidelines | 1496 (80) | 158 (76) | 0.105 |

n: number; IQR: 25-75 interquartile range; PSI: pneumonia severity index; ICU: intensive care unit; CXR: chest radiograph; ESBL: extended-spectrum beta-lactamase; MDR: multidrug resistant (including methicillin-resistant *S. aureus*, *Pseudomonas aeruginosa* resistant to antipseudomonal penicillins, cephalosporins, carbapenems, and quinolones, *Stenotrophomonas maltophilia*, vancomycin-resistant *Enterococcus*, *Acinetobacter baumanii*, extended spectrum b-lactamase producing *Enterobacteriaceae,* and other non-fermenting Gram-negative bacilli); SpO_2_: oxygen saturation; PaO_2_: partial pressure of oxygen in arterial blood; PaCO_2_: partial pressure of carbon dioxide in arterial blood; ERS: European Respiratory Society. ^+^Hypotension defined as Systolic blood pressure <90 mm Hg or diastolic blood pressure < 60 mm Hg. Differences in continuous variables were monitored with Mann-Whitney U Test ; differences in proportions were monitored with the chi square test.

The meta-analysis of the absolute risk difference in mortality between the study groups failed to exhibit a significant heterogeneity by centers (after accounting for multiplicity of comparisons, which reduced the critical p to 0.025): I^2^=0% (p=0.595) for the comparison of ARF vs. controls, and I^2^=68.2% (p=0.043) for the comparison of ARF and SS vs. controls, see eFigure 1.
