# Supplementary material for: Phenotyping community-acquired pneumonia according to the presence of acute respiratory failure and severe sepsis
Source: Respir Res. 2014 Mar 4;15(1):27. doi: 10.1186/1465-9921-15-27 (PMC4015148; doi:10.1186/1465-9921-15-27)

**Additional file 2: Figure S1. The meta-analysis of the absolute risk difference in mortality between the study groups. ARF: acute respiratory failure, SS: severe sepsis**


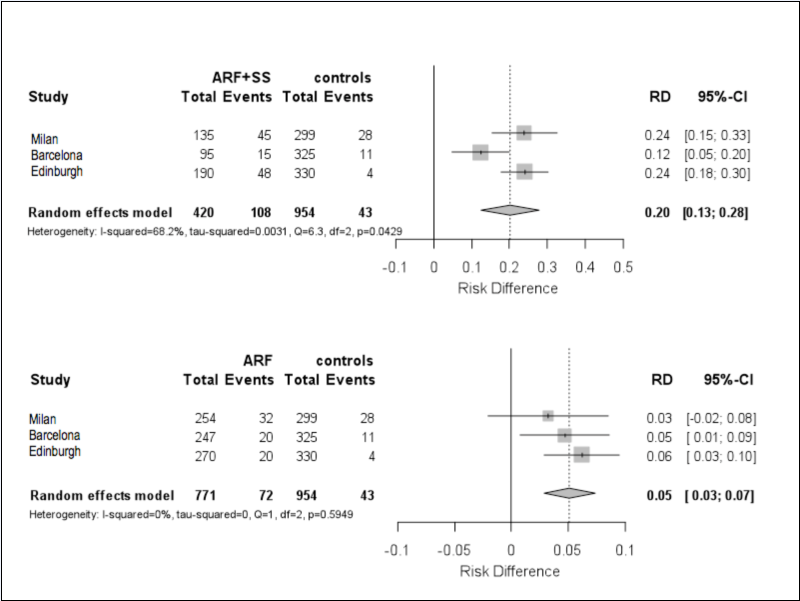

Supplement: Additional file 2: Figure S1 — The meta-analysis of the absolute risk difference in mortality between the study groups. ARF: acute respiratory failure, SS: severe sepsis. [file 1465-9921-15-27-S2.doc]
